# Fungemia associated with *Schizophyllum commune* in Brazil

**DOI:** 10.1371/journal.pntd.0005549

**Published:** 2017-06-29

**Authors:** Manoel Marques Evangelista Oliveira, Alberto Santos Lemos, Marcelo Luiz Carvalho Gonçalves, Rodrigo Almeida-Paes, Vitor Ribeiro Gomes de Almeida Valviesse, José Alfredo Moreira, Marco Antônio Sales Dantas Lima, Eleonora Carregal, Maria Clara Gutierrez Galhardo, Cristiane da Cruz Lamas, Rosely Maria Zancopé Oliveira

**Affiliations:** 1 Laboratório de Micologia, Instituto Nacional de Infectologia Evandro Chagas, Fundação Oswaldo Cruz, Rio de Janeiro, Rio de Janeiro, Brazil; 2 Programa de Residência Médica em Infectologia, Instituto Nacional de Infectologia Evandro Chagas, Fundação Oswaldo Cruz, Rio de Janeiro, Rio de Janeiro, Brazil; 3 Centro de Clínicas, Instituto Nacional de Infectologia Evandro Chagas, Fundação Oswaldo Cruz, Rio de Janeiro, Rio de Janeiro, Brazil; 4 Laboratório de Pesquisa Clínica em Neuroinfecções, Instituto Nacional de Infectologia Evandro Chagas, Fundação Oswaldo Cruz, Rio de Janeiro, Rio de Janeiro, Brazil; 5 Serviço de Imagem, Instituto Nacional de Infectologia Evandro Chagas, Fundação Oswaldo Cruz, Rio de Janeiro, Rio de Janeiro, Brazil; 6 Laboratório de Pesquisa Clínica em Dermatologia Infecciosa, Instituto Nacional de Infectologia Evandro Chagas, Fundação Oswaldo Cruz, Rio de Janeiro, Rio de Janeiro, Brazil; University of Tennessee, UNITED STATES

## Case report

**Fig 1:**
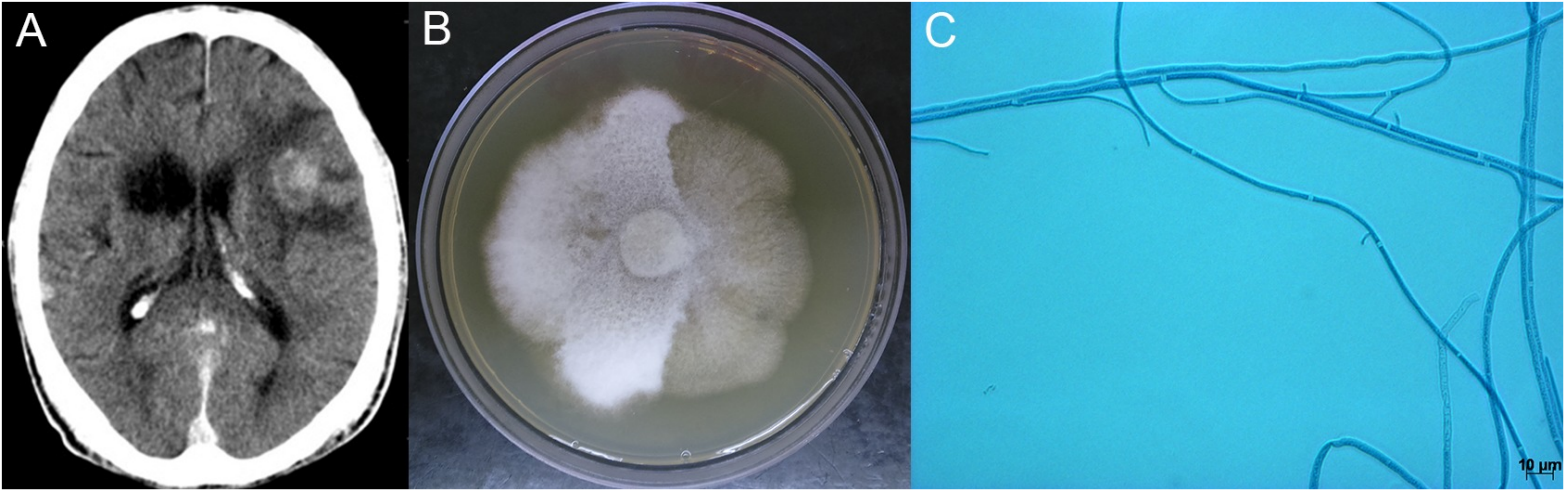
*Schizophyllum commune* infection in an HIV-infected patient. **(A)** Brain CT scan showing contrast-enhancing lesions in both cerebral hemispheres associated with mass effect and a hypodense lesion in the right caudate nucleus suggestive of encephalomalacia from previous lesions. **(B)** *S*. *commune* 1083H colony on potato dextrose agar (PDA) agar after 14 days at 25°C; fruiting bodies are not present on the hyphae. **(C)** Slide culture of the *S*. *commune* isolate on PDA showing hyaline, septate hyphae with spicules and without clamp connections.

Fungi have been reported since the 1980s in patients with HIV/AIDS as primary drivers for mortality in this population [1, 2]. *Schizophyllum commune* is a fungus uncommonly described in humans because of the difficulties encountered in the laboratory identification of this agent [3]. This fungus is ubiquitous and grows on trees and decaying wood, being widely distributed in the environment [4]. Infection occurs by inhalation of the basidiospores, with bronchopulmonary disease and sinusitis accounting for 94% of cases. Extrapulmonary dissemination was described and the brain was the most affected organ, manifesting as brain abscess [5]. In HIV-infected patients, manifestations were related to chronic sinusitis [3, 6]. Herein, we report the first case of bloodstream infection with *S*. *commune* in an HIV-infected patient. A 49-year-old Brazilian man who had received HIV diagnosis a few days before was admitted to our hospital with mild dyspnea, chronic productive cough, weight loss, headache, and fever. The diagnosis of pulmonary tuberculosis was ascertained by direct examination of sputum samples for acid-fast bacilli (AFB) and treatment was started with rifampicin, isoniazid, ethambutol, and pyrazinamide. The patient was antiretroviral therapy (ART) naïve, with a baseline total CD4+ lymphocyte (TCD4+) count of 106 cells/μl and an HIV viral load (VL) of 180,990 copies/ml (5.3 log10). He underwent a brain computed tomography (CT) scan and lumbar puncture (LP) due to headache and neck stiffness found on physical examination. The initial cerebrospinal fluid (CSF) analysis revealed 166 cells/mm^3^ (95% mononuclear), protein 71.7 mg/dl, normal glucose levels, and negative direct microscopy for fungi, AFB, or other bacteria. Cultures were negative after appropriate incubation periods. The brain CT scan revealed a hypodense lesion in the right caudate nucleus suggestive of encephalomalacia from a previous lesion. Steroids were added to the antituberculous regimen with the consideration of tuberculosis meningitis, and the patient was discharged. ART was initiated in the outpatient unit with zidovudine, lamivudine, and efavirenz. After 2 weeks, the patient was readmitted to the hospital with severe headache, disorientation, and paraparesis. A new CT scan showed several new contrast-enhancing lesions located in both cerebral hemispheres (Fig 1A), associated with mass effect. A new LP revealed an inflammatory pattern similar to that observed in the previous admission. The temporal associations of these abnormalities with ART introduction led to the presumptive diagnosis of central nervous system immune reconstitution inflammatory syndrome (CNS-IRIS). Two days after this second admission, the mycology laboratory concluded the identification of a rare fungus that had grown after 10 days in blood cultures (Bact/Alert R FA Plus) performed during the patient’s previous hospitalization. Amphotericin B deoxycholate (1 mg/kg/day) was started, but the patient’s neurological status further deteriorated and he was transferred to intensive care unit (ICU). Cotrimoxazol was also started empirically for toxoplasmosis. The thermotolerance test at 37°C with the fungus isolated from the blood culture revealed a fast-expanding, cottony white mycelium. Subcultures in potato dextrose agar (PDA) yielded after a week a cottony white colony that turned light grey (Fig 1B) with a distinctive fruity odor, which appeared as hyaline, septate, nondichotomously branching hyphae in a lactophenol cotton blue mount (Fig 1C). Since it was not possible to achieve fungal identification only with conventional mycological techniques, partial sequencing of the internal transcribed spacer (ITS) region of ribosomal DNA (rDNA) was performed using ITS1 (TCCGTAGGTGAACCTGCGG) and ITS4 (TCCTCCGCTTATTGATATGC) primers [7] and an annealing temperature of 58°C. Automated sequencing was done using the Sequencing Platform at Fundação Oswaldo Cruz, Brazil [8]. Sequences were edited with Sequencher 4.9 software and compared by BLAST with sequences available from NCBI/GenBank, and 99% concordance with sequences of *S*. *commune* deposited there was found (LN7146, AF280759). The ITS sequence has been deposited in GenBank under accession number KU255858. After 1 month, the patient gradually improved, and his CD4 count increased to 424 cells/μl. Follow-up brain CT as well as CSF analysis showed improvement of the inflammatory pattern. After 7 months of treatment with amphotericin B, fluconazole 200 mg/day was given as maintenance therapy because of its better penetration through the CSF barrier. The patient remains well after 3 years of follow-up. HIV immunodeficiency can change the medical course of diseases and lead to rare opportunistic manifestations. We highlight the systemic involvement of the fungus, and, as no brain biopsy was performed, we can only speculate that it was a possible cause of the brain abscess. This is the first report of a patient with fungemia due to *S*. *commune* who survived and made a good recovery after antifungal therapy.

## Ethics statement

This work was approved by the Research Ethics Committee (CEP) Fiocruz, CAAE:28063114.2.0000.5262. The patient has signed the consent form for publication.

**Key learning points**

- *Schizopyllum commune* can cause bloodstream infection in HIV-infected patients.
- *S*. *commune* identification needs molecular approaches, such as internal transcribed spacer (ITS) sequencing.
- Treatment with amphotericin B deoxycholate and fluconazole showed to be effective, leading to patient survival.
